# FGFF Descriptor and Modified Hu Moment-Based Hand Gesture Recognition

**DOI:** 10.3390/s21196525

**Published:** 2021-09-29

**Authors:** Beiwei Zhang, Yudong Zhang, Jinliang Liu, Bin Wang

**Affiliations:** 1 School of Information Engineering, Nanjing University of Finance and Economics, Nanjing 210023, China; liujinliang@vip.163.com (J.L.); wangbin@nufe.edu.cn (B.W.); 2 School of Computing and Mathematical Sciences, University of Leicester, Leicester LE1 7RH, UK; yudongzhang@ieee.org

**Keywords:** FGFF descriptor, Hu moment invariants, finger thickness, hand gesture recognition, weighted AdaBoost classifier

## Abstract

Gesture recognition has been studied for decades and still remains an open problem. One important reason is that the features representing those gestures are not sufficient, which may lead to poor performance and weak robustness. Therefore, this work aims at a comprehensive and discriminative feature for hand gesture recognition. Here, a distinctive Fingertip Gradient orientation with Finger Fourier (FGFF) descriptor and modified Hu moments are suggested on the platform of a Kinect sensor. Firstly, two algorithms are designed to extract the fingertip-emphasized features, including palm center, fingertips, and their gradient orientations, followed by the finger-emphasized Fourier descriptor to construct the FGFF descriptors. Then, the modified Hu moment invariants with much lower exponents are discussed to encode contour-emphasized structure in the hand region. Finally, a weighted AdaBoost classifier is built based on finger-earth mover’s distance and SVM models to realize the hand gesture recognition. Extensive experiments on a ten-gesture dataset were carried out and compared the proposed algorithm with three benchmark methods to validate its performance. Encouraging results were obtained considering recognition accuracy and efficiency.

## 1. Introduction

Hand gestures carry rich information and provide a natural yet important method for different people to interact in their daily life. They have been used as a friendly interface between humans and computer systems, which enables an intuitive and convenient human–computer interaction, and have found many applications in natural human–computer interaction, such as intelligent robot control, smart homing, virtual reality, computer games, and some quietness-required environments. In [1], the authors explored the recognition application of handwritten Arabic alphabets by tracking and modeling the motion of the hand. To this end, recent years have witnessed an active research interest in the field of hand gesture recognition and human action recognition.

Traditional vision-based recognition algorithms mainly utilize the information of color or texture from 2D RGB camera, which is typically affected by external environments such as illumination, skin color, and cluttered background. Their limitation is the loss of 3D structure information, which obviously decreases their robustness and accuracy. In order to improve the robustness and simplify the hand localization and segmentation, some researchers suggested the use of a colored glove or black belt on the wrist of the gesturing hand [2]. Furthermore, accelerometers, magnetic trackers, and data gloves are involved in obtaining the three-dimensional information of gesture for easy image processing and 3D motion capturing at the granularity of the fingers. However, these strategies are only suitable for handling some simple gestures. When the gesture becomes more complex, it will obviously reduce the recognition accuracy. Furthermore, it impedes the invisibility of the interface for the users and brings increased inconvenience and can be cumbersome in some cases in which many cables may be involved [3].

Thanks to the development of inexpensive depth cameras, e.g., the Kinect sensor, a new and desirable method is provided to extract motion and visual information for human activities. Instead of wearing data gloves or any other auxiliary equipment, the gesturing hand can be detected and segmented efficiently with the Kinect sensor. Therefore, more and more research has paid attention to this platform in recent years, and the authors can be referred to [4,5] for a comprehensive review work. Basically, all of the existing algorithms can be classified into two categories, i.e., skeleton-based algorithms and depth-based algorithms, depending on the types of input data. The former uses 3D coordinates of the joints to represent the model of full human body. The method proposed by Thanh and Chen [6] falls into this type, which extracted the discriminative patterns as local features to classify skeleton sequences in human action recognition and the key frames were constructed based on skeleton histogram. Many other works tried to study the spatial-temporal descriptions from Kinect skeleton data, e.g., the angular representation [7] and skeletal shape trajectories [8]. As the skeleton information carries little details and is only suitable for human body tracking, it is difficult to detect and segment a small object, such as a human hand, which occupies a very small portion of the image with more complex articulations [9]. In practice, this type of work also suffers from contour distortions since little noise or slight variations in the contour would severely perturb the topology of its skeletal representation.

On the other hand, depth-based algorithms employ depth information for action recognition which shows its advantages in many situations. Joongrock and Sunjin [10] propose an adaptive local binary pattern from depth images for hand tracking. There are some researchers apply the dynamic time warping algorithm for hand gesture recognition with the extracted finger lets, stroke lets, or other characteristics from its depth information [11]. Their work shows that a concise and effective feature descriptor is critical for the recognition performance. Kviatkovsky [12] and Chang [13] suggest the use of covariance descriptors to encode the statistics of temporal shape and motion changes in a low dimensional space with an efficient incremental update mechanism. Zhang and Yang et al. [14] presented a low-cost descriptor via computing 3D histograms of textures from a sequence of depth maps. In their work, the depth sequences were first projected onto three orthogonal Cartesian plane to form three projected maps, then the sign-based, magnitude-based and center-based descriptor salient information were extracted, respectively. Similarly in Reza [15], the weighted depth motion map was proposed to extract the spatiotemporal information by an accumulated weighted absolute difference of consecutive frames and the histogram of gradient and local binary pattern were exploited for the feature descriptor.

Despite many algorithms and solutions in applying the Kinect for hand gesture and action recognition, it still is an open problem in practical applications considering the robustness, accuracy, and computational complexity. As the above-reviewed algorithms cannot process nonlinear and high dimensional data, some researchers tried to solve this problem via the recent advances in convolutional neural network [16,17,18,19]. The advantage of the deep neural network lies in that it is able to automatically extract hierarchical features to hold more abstract knowledge from video sequences and thus reduce the need for feature engineering. However, it requires a long time to train and a huge amount of labeled training data, which may not be available in some cases. For small human action recognition datasets, the deep learning methods may not provide satisfactory performance. The extracted features lack of specific physical meaning, thus it is difficult to analyze their characteristics.

It is known that the hand gesture delivers its meaning by the movement of a hand. Different hand gestures are mainly differentiated by the postures of the fingers. When the fingers display different postures, their contour shapes can be differentiated clearly. Therefore, many researchers focus on the extraction of various features [20,21,22,23,24,25,26,27]. Ren et al. [23] employed time series curves to characterize the Euclidean distance between the hand contour and the palm center, where the starting point of the curve is not easy to track without any auxiliaries. Huang and Yang in [24] suggested a multi-scale descriptor including area of major zone, length of major segment, and central distance. In their method, it is important to choose a proper scale number and a starting point to align all points on the shape contour. Wang [25] constructed features with peak values and valley values from the trend of slope difference distribution of the contour points. The robustness and accuracy for extracting the peak and valley values are prone to be disturbed by various noise. Multiple types of features such as the rotation of joints and fingertip distances were proposed in [26], where the positions of 20 joint points were required to extract from the depth map according to the characteristics of the hand model. Obviously, their computational complexity is high and the extraction accuracy is not easy to control. In practice, it is desired that the feature descriptor possesses the properties of scale, translation, and rotation invariants [28,29]. For example, the contour of the hand region was extracted with Moore neighbor algorithm and the convex hull by Graham scan algorithm, and then the Hu moment invariants for hand gesture recognition were estimated in [28]. However, this algorithm is sensitive to noise and the computational load is heavy.

Basically, the major problem in the surveyed methods lies in that the features representing those gestures are not sufficient, which leads to poor performance and weak robustness. Therefore, this work aims at a comprehensive and discriminative feature for hand gesture recognition. Here, a new framework for hand gesture recognition is proposed by combing Fingertip Gradient orientation and Finger Fourier (FGFF) descriptor together with the modified Hu moments using the depth information collected by a Kinect sensor, where the former concentrates on the details of fingers and the latter encodes the structure of hand contour. According to the characteristics of hand depth image, two efficient procedures are suggested to segment the hand and extract fingers. Taking 10 types of hand gestures representing the digital numbers from zero to nine as an example, a weighted AdaBoost classifier is constructed based on the finger-earth mover’s distance (FEMD) method and SVM model. Extensive experiments on a ten-gesture dataset collected in our lab were carried out to validate the proposed algorithm. Compared with three benchmark methods, our work achieves a better performance in terms of recognition accuracy, robustness and computational complexity (a 96.6% mean accuracy on the challenging 10-gesture dataset with average 0.05 s per frame).

The remainder paper is structured as follows. Two algorithms for the hand region segmentation and finger extraction are discussed in Section 2. Section 3 elaborates the FGFF descriptor and modified Hu moments. The weighted AdaBoost classifier for hand gesture recognition is introduced in Section 4. Section 5 presents some experimental results and analysis. Finally, this paper is concluded briefly in Section 6.

## 2. Hand Segmentation and Finger Extraction

**Figure 1:**
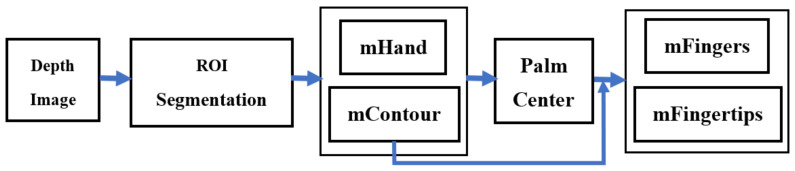
The flowchart of the hand segmentation and finger extraction.

This section firstly elaborates the technique for hand segmentation to obtain the interior points (mHand) and contour points (mContour) of a hand, then suggests two algorithms for extracting the palm center, fingers, and fingertips, denoted as mFingers and mFingertips, respectively. The flowchart of the hand segmentation and finger extraction from its depth image can be summarized in Figure 1.

### 2.1. Hand Region Segmentation

In order to effectively segment the hand region from its depth image, Ren et al. [23] suggested wearing a black belt to highlight the boundaries between the hand and the wrist. It is well known that the gray value of each pixel in the depth image represents the distance between the point and the sensor. The smaller the value is, the closer the distance becomes, and vice versa. Without loss of generality, it can be assumed that the hand is located in the front of the body when performing the gesture and there are no obstacles between the Kinect sensor and the performer. Therefore, the distance between the hand area and the sensor is the closest in this scenario. Considering that there is a certain range of hand size for general adults, this paper proposes a double-threshold-based region growing method to realize the segmentation of hand region and enable a natural mode of interaction without wearing any auxiliary object.

**Figure 2:**
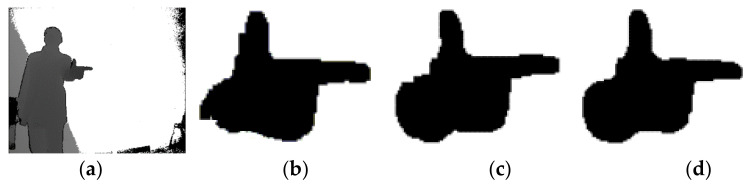
Different effects of the good and bad threshold values in segmentation: (**a**) gives the depth image while (**b**–**d**) show different segmentation results using different threshold values.

Firstly, the nearest point to the Kinect sensor denoted as $M_{min}$, is searched in the depth image. With $M_{min}$ as a seeding point, the eight-neighborhood region growing method is iteratively performed. Here, we set three iterative conditions for the growing point as: (1) this point has not been grown before; (2) its difference with the depth value of the preceding point is less than the threshold value of Th1; (3) the difference with the average value of the point set that has been grown is less than Th2. When the iteration process ends, the proper hand region is obtained as

(1) $$H=\left\{M_{x,y}|d_{M_{min}}<d_{M_{x,y}}<d_{M_{min}}+d_{th}\right\}\;s.t.\;\left|area\left(H\right)-A_{0}\right|<A_{th}$$

where $d_{M_{x,y}}$ and $d_{M_{min}}$, respectively, denote the depth value of the point $M_{x,y}$ and $M_{min}$ in the depth image, while $d_{th}$ represents the depth range of the detected hand region considering the general size of human hand. $A_{0}$ and $A_{th}$ denote the area of average hand region and its range estimated from the training dataset who helps to remove the contamination regions or fake hand regions from the depth image. The parameter Th1 is used to keep consistency and smoothness in the ROI while Th2 decides whether oversegmentation is involved or not. Their values are set empirically and used to ensure the local and global consistency when growing the hand region. Our experimental results show that some holes may exist when the value of Th1 is set too high or the value of Th2 is too low. Oversegmentation will happen for a larger value of Th2, e.g., part of the wrist may be included as the hand region if a larger Th2 is used. Satisfactory results are obtained when Th1 ranges from 3 to 4 and Th2 ranges from 8 to 10. Figure 2 shows different effects of various threshold values in the hand region segmentation. Here, Th1 and Th2 are, respectively, set 5 and 7 in Figure 2b, while the empirical instructions are followed for the thresholds in Figure 2c,d. Obviously, better results are obtained in the latter two cases. This observation is critical where oversegmentation is needed to obtain part of the wrist as an anchor point to regularize the local features in the next section.

Compared with the traditional threshold-based segmentation, the advantage of this mechanism is that the boundary of the hand region is relatively smooth and there are fewer holes, as will be shown later. Therefore, it is easy for subsequent processing. A point is deemed as the interior point if its 3 × 3 neighborhood is also in the hand region, otherwise it is the contour point. In this manner, the hand region H can be divided into interior point set and contour point set, respectively, denoted by mHand and mContour.

### 2.2. Extraction of Palm Region

In general, the area of the palm as well as its roundness is larger than those of the fingers for any hand gesture. Based on this observation, the palm region and its center can be found mathematically as the largest inscribed circle in the hand region. Initially, the palm center and the maximum radius of inscribed circle are assumed as $M_{0}$ and $R_{0}$. The process for the solution can be summarized as Algorithm 1.
**Algorithm 1** Calculating the center and maximum radius of the palm regionInput: Interior point set mHand and contour point set mContour Output: The radius $R_{0}$ and center of the palm center $M_{0}$ Begin  Step1: Set $R_{0}=0$ and $M_{0}=[]$ initially.   Step2: For one point in mHand, compute its distances from all the points in mContour.  Step3: Find the minimum distance value, update it as $R_{0}$ and the corresponding point as $M_{0}$ if it is larger than $R_{0}$.   Step4: Go to Step2 and repeat until all the points in mHand are iterated.   Step5: Finally, the $R_{0}$ and $M_{0}$ are obtained as the radius of the inscribed circle of the palm region and its center. End

### 2.3. Fingertip Extraction

When performing a gesture, different meanings are conveyed by different finger shapes and their relative positions, thus representing different digital gestures. It is obvious that the fingertip is the point farthest from the palm center in the contour point set. With this observation, the average distance between the palm center and those points in the contour point set is used to limit the scope of the fingertip and finger extraction to reduce the computational complexity, which means that those points within the average distance will be ignored. Assuming that all the fingers and fingertips are, respectively, denoted as mFingers and mFingertips, the proposed solution is briefly summarized as Algorithm 2.
**Algorithm 2** Extraction of fingertips and fingersInput: The palm center $M_{0}$ and contour point set mContour Output: The mFingertips and mFingers Begin Step1: Initialize the sets of candSet, mFingertips and mFingers;  Step2: Calculate the average distance $R_{avg}$ from $M_{0}$ to all the points in mContour;   Step3: Add those points to candSet, if their distances from $M_{0}$ greater than or equal to $R_{avg}$;   Step4: For each of the elements in candSet, compute its distance from $M_{0}$. Then find the one corresponding to the largest distance and move it to both mFingertips and mFingers, and move all the rest points that are connected with it in the candSet to mFingers;   Step5: Go to Step4 and repeat until candSet is empty;   Step6: The fingertips and fingers are obtained in mFingertips and mFingers. End

**Figure 3:**
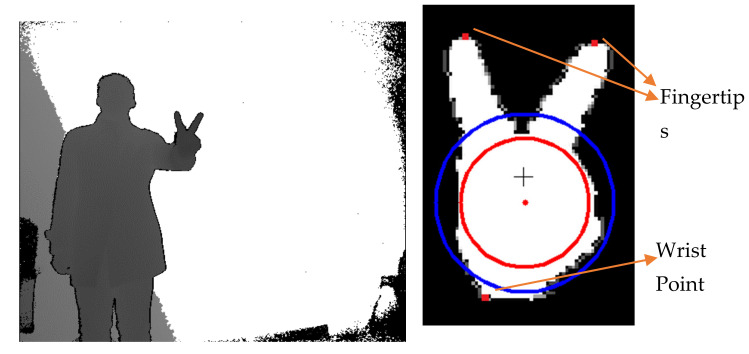
The depth image (**left**) and extracted elements (**right**): palm center with a higher quality denoted by RED point versus central moment of the hand by BLACK cross where the red and blue circles, respectively, represent inscribed and averaging circles.

Figure 3 shows the original depth image and its segmented hand region. The palm center and inscribed circle extracted with the first algorithm are referred to the red dot and circle in the right figure. For comparison, the central moment of the hand region is estimated and denoted as black cross. Obviously, the extracted palm center has a higher quality than the central moment. The blue circle shows the average distance between the palm center and the contour point set. From this figure, it is observed that the fingertips, fingers, and wrist of this hand gesture can be easily obtained with the second algorithm.

On the whole, it can be seen that the hand area, contour, palm center, and fingertips are correctly extracted, and their boundaries are fairly smooth and graceful. It is worth to note that the wrist should be extracted as one finger with the above algorithm and its remote point as one fingertip since oversegmentation is involved, as discussed in Section 2.1. We will show how to identify it next.

The wrist can be determined considering that the wrist visually has the largest thickness compared with the fingers. Let the average circle of hand region be denoted as mAvgcircles. Mathematically, the remote point in the wrist can be defined as

(2) $$\begin{array}{l} M_{wr}=\left\{M|M\in mFingertips\right\}\\ \;s.t.{\;\mathrm{argmax}}_{M_{1},M_{2}\in mFingers\cap mAvgcircles}dist\left(M_{1},M_{2}\right) \end{array}$$

where $M_{1}$ and $M_{2}$ are two elements in mFingers associated with M and $dist\left(M_{1},M_{1}\right)$ represents their Euclidean distance. Once the wrist point is found, it can be used as a benchmark for ordering the point sequences of the fingers since it is stationary for hand gestures. Finally, the fingertips and their related fingers can be easily identified.

## 3. FGFF Descriptor and Hu Moments

### 3.1. FGFF Descriptor

The FGFF descriptor consists of fingertip-emphasized and finger-emphasized components, i.e., the fingertip gradient orientations and finger Fourier descriptor. Let the palm center $M_{0}$ be $\left[x_{p},y_{p},d_{p}\right]$ with $x_{p}$ and $y_{p}$ as image coordinates and $d_{p}$ its depth value. Similarly, the i-th fingertip in mFingertips can be expressed as $M_{fi}=\left[x_{fi},y_{fi},d_{fi}\right]$. The gradient orientation can be constructed for each fingertip

(3) $$FG_{i}={\left(x_{i},y_{i},d_{i}\right)}^{T}$$

where $x_{i}=x_{fi-}x_{p}$, $y_{i}=y_{fi-}y_{p}$, and $d_{i}=d_{fi-}d_{p}$. The relative position is used here to eliminate the differences from different performers and avoid the distortions of some gestures. In Equation (3), the descriptor encodes the position and the depth value, as well as the orientation information for each fingertip, which is invariant to translation, rotation, and scale transformation with normalization.

On the other hand, the finger part associated with each fingertip is obviously connected, which can be represented as a point sequence. Let $s=\left\{x\left(k\right),\;y\left(k\right)|k=0,\;1,\;2,\cdots ,L\right\}$ be the *i*-th finger. In complex space, it is formulated as 1-D problem

(4) $$s\left(k\right)=x\left(k\right)+jy\left(k\right)\;s.t.\;j^{2}=-1$$

**Figure 4:**
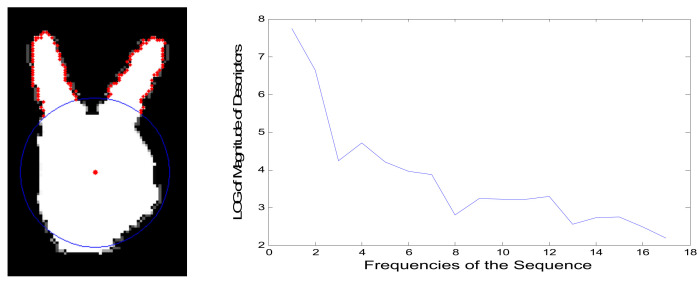
The fingers and distribution of FF descriptor vs. frequencies.

With 1-D discrete Fourier transform, the spectrum in frequency domain is obtained. The left and right figures in Figure 4, respectively, give the point sequences of fingers and distribution of FF descriptor. It is observed that its energy is mainly concentrated with the lower frequencies and decreases rapidly with the increasing frequency. In this work, we find that more than 80 percent of energy is carried by the first seventeen magnitudes. Therefore, the Fourier descriptor denoted as $FF_{i}$, is assigned, considering a small range of lower frequencies. Finally, the FGFF descriptor for the finger can be constructed with $FG_{i}$ and $FF_{i}$.

### 3.2. Modified Hu Moments

The Hu moment is an important feature used to describe image shape based on moment transform. The invariants of translation, rotation, and scale are preserved by the operations of centralization and normalization of the Hu moments for continuous functions. In gesture recognition, there are differences in action amplitude, hand size, and relative positions with respect to the sensor among different performers. Therefore, Hu moment invariants show unique advantages in such cases and can be used to encode the contour of a whole hand. However, different from the continuous function, the hand region is extracted as discrete data. We will first show that the Hu moments do not satisfy the scale invariant and then provide our modification in what follows.

Supposing the performer executes the same hand gesture at two different positions successively, the point coordinates on his hand in depth image will change from $\left(x,\;y\right)$ to $\left(x^{\prime },y^{\prime }\right)$. Let the scale factor be *ρ*, then $x^{\prime }=\rho x$, $y^{\prime }=\rho y$. According to the definition of moment, we have

(5) $$x^{\prime }-x_{c}^{\prime }=\rho x-\frac{\sum_{x,y}\rho xf\left(x,y\right)}{\sum_{x,y}f\left(x,y\right)}=\rho \left(x-x_{c}\right)$$

and similarly

(6) $$y^{\prime }-y_{c}^{\prime }=\rho \left(y-y_{c}\right)$$

Then we get

(7) $$\mu_{pq}^{\prime }=\sum_{x,y}{\left(x^{\prime }-x_{c}^{\prime }\right)}^{p}{\left(y^{\prime }-y_{c}^{\prime }\right)}^{q}f^{\prime }\left(x^{\prime }-y^{\prime }\right)=\rho^{p+q}\mu_{pq}$$

According to the normalization formula of centralized moment, we obtain

(8) $$\emptyset_{pq}^{\prime }=\frac{\mu_{pq}^{\prime }}{\mu_{00}^{\prime r+1}}=\rho^{p+q}\emptyset_{pq}$$

where $r=\frac{p+q}{2}$. It can be seen from the above formula that the normalized central moment of discrete data is a function of the scale factor and the power of the moments. As a result, the Hu moment is no longer scale invariant. To eliminate the scale factor, a group of new formula of Hu moments is suggested here:

(9) a1=lgh1h02a2=lgh2h0h1a3=lgh3h0h1a4=lgh4h2h3a5=lgh5h0h2a6=lgh6h4

where $h_{0}$, $h_{1}$, ⋯, $h_{6}$ represent functions of $\emptyset_{pq}^{\text{’}}$.

Compared with the forms of Hu moment with sixth power reported in the literature [28], the power degree in Formula (9) is much lower and hence has higher robustness to noise. In this manner, a 6-D Hu moment descriptor denoted as $H_{u}$ can be formulated for each gesture from its depth image, which is also invariant to translation, rotation, and scale transformation.

## 4. Weighted AdaBoost Classifier

As shown in the above section, the number of extracted local finger features may be different when performing different types of hand gestures. Therefore, the finger-earth mover’s distance (FEMD) method is used to estimate the similarity between two gesture images with their FGFF descriptors. For the modified Hu moment invariant features, this work employs a support vector machine to train and test those gesture images. Finally, the recognition results of these two methods are merged together with the weighted AdaBoost Classifier to perform the gesture recognition algorithm.

### 4.1. The Finger-Earth Mover’s Distance

The finger-earth mover’s distance method originated from the classical transportation problem and updated by Ren et al. [23] as FEMD. Let $R_{c}$ refer to the FGFF descriptor extracted from an arbitrary hand gesture depth image with *c*th category in the training dataset. Let *T* represent the testing hand gesture, whose category can be determined by the category of the training sample with the highest similarity, which is defined as:

(10) $$c^{*}=argmin_{c}\min\{FEMD(R_{c},T)|R_{c}\;is\;a\;sample\;in\;category\;c\}$$

where the parameter *c* ranges over all categories and $c^{*}$ denotes the category corresponding the training sample with the highest similarity.

Suppose there are *m* fingers in the mFingers set for a training sample and its local feature descriptor can be represented as $R_{c}=\left\{\left(r_{1},w_{r_{1}}\right),\cdots ,\left(r_{m},w_{r_{m}}\right)\right\}$, where $r_{i}$ and $w_{r_{i}}$, respectively, denotes the *i*-th finger and its weight factor. In the same manner, the feature descriptor for a testing hand gesture with *n* fingertips is denoted as $T=\left\{\left(t_{1},w_{t_{1}}\right),\cdots ,\left(t_{n},w_{t_{n}}\right)\right\}$.

Let $D=\left[d_{ij}\right]$ be the distance matrix between $R_{c}$ and *T*, in which its element can be computed as $d_{ij}=r_{i}-t_{j}{}_{2}$. Their FEMD distance is defined as the least work moving the earth piles from $R_{c}$ to *T* plus the penalty on the empty hole that is not filled with earth

(11) $$\begin{array}{l} FEMD\left(R_{c},T\right)=\beta E_{move}+\left(1-\beta \right)E_{empty}\\ \;=\frac{\beta \sum_{i=1}^{m}\sum_{j=1}^{n}d_{ij}f_{ij}+\left(1-\beta \right)\left|\sum_{i=1}^{m}w_{r_{i}}-\sum_{j=1}^{n}w_{t_{j}}\right|}{\sum_{i=1}^{m}\sum_{j=1}^{n}f_{ij}} \end{array}$$

where the element $f_{ij}$ in matrix *F* represents the workload of transporting $r_{i}$ to $t_{j}$ and $\sum_{i=1}^{m}\sum_{j=1}^{n}f_{ij}$ as the normalization factor. The parameter β modulates the importance between $E_{move}$ and $E_{empty}$. The sensitivity of this parameter to the recognition algorithm had been discussed in [23] and showed that the best results could be obtained when β falls in the range of 0.3 and 0.6. As the FEMD depends on the matrix *F*, its objective function and constrains is given as what follows

(12) $$F=argminWORK\left(R,T,F\right)=argmin\overset{m}{\underset{i=1}{\sum }}\overset{n}{\underset{j=1}{\sum }}d_{ij}f_{ij}\;s.t.\left\{\begin{array}{c} f_{ij}\geq 0\;i=1,\;\cdots ,m;\;j=1,\;\cdots ,n\\ \overset{m}{\underset{i=1}{\sum }}w_{r_{i}}\leq w_{t_{j}}\\ \overset{n}{\underset{i=1}{\sum }}w_{t_{j}}\leq w_{r_{i}}\\ \overset{m}{\underset{i=1}{\sum }}\overset{n}{\underset{j=1}{\sum }}f_{ij}=min\left(\overset{m}{\underset{i=1}{\sum }}w_{r_{i}},\overset{n}{\underset{i=1}{\sum }}w_{t_{j}}\right) \end{array}\right.$$

In this manner, the finger-earth mover’s distances of the testing gesture with all the training samples are obtained. According to Formula (10), the category of the gesture is assigned to that of the training sample corresponding to the minimum finger-earth mover’s distance. It should be noted that the proposed FGFF descriptor used here is completed as it encodes the relative position information of one fingertip, palm center, and wrist point, as well as the structure of the finger.

### 4.2. Support Vector Machine

Support vector machine (SVM) is a generalized linear classifier based on supervised learning for binary classification on the testing data. For multi-class problems, there are various deformations using SVM, e.g., one-to-one method, one-to-remainder method, and binary-tree method. Among those, the one-to-one method has the characteristics of high correct recognition rate, simplicity, and efficiency. Its basic idea is: Given an N-class classification problem, firstly training $\frac{N\left(N-1\right)}{2}$ support vector machines, and then the classification result of the testing data can be determined by voting principle with all the SVMs. For the recognition of 10 kinds of digital hand gestures in this paper, the one-to-one method is employed and hence a total of 45 support vector machines are trained, then the classification results are statistically analyzed and fused with those from Section 4.1. Finally, the category of the testing data could be determined.

### 4.3. Weighted AdaBoost Classifier (WAC)

The FGFF descriptor and modified Hu moment invariants are deemed as local detailed features and global structural features extracted from the hand gesture depth images. The finger-earth mover’s distance method and support vector machine model are used as the base classifiers to recognize the hand gesture with those features. During training, the performance of each of the classifiers can be obtained with the training set. Given one test sample, say $T_{i}$, its category can be decided with the following weighted AdaBoost classifier

(13) $$Label\left(T_{i}\right)=\sum \alpha_{j}G_{j}\left(T_{i}\right)$$

where $G_{j}\in${FEMD method, SVM model} is the *j*-th basis classifier with the weighted factor

(14) $$\alpha_{j}=\frac{1}{2}log\frac{P\left(G_{j}\left(R_{i}\right)=Label\left(R_{i}\right)\right)}{1-P\left(G_{j}\left(R_{i}\right)=Label\left(R_{i}\right)\right)}$$

As can be seen from the above equation, the weighted factor $\alpha_{j}$ is an increasing function of the recognition accuracy of a base classifier. When the recognition accuracy is more than 50%, we have $\alpha_{j}>0$. With the increasing accuracy, its relative role in the AdaBoost classifier becomes more and more important. In this manner, we highlight the excellent classifier in our algorithm. Our experiments also show that the accuracy and stability will be strengthened by constructing the combination model with such an addition mechanism.

## 5. Experiments and Analysis

This section presents our experimental results on the ten-gesture dataset collected in our lab to validate the proposed hand gesture recognition algorithm. Some details and insights in the algorithm are discussed together with comparison with three benchmark methods to demonstrate the improvement of our algorithm.

### 5.1. Experimental Dataset

**Figure 5:**
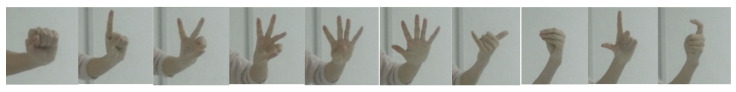
The predefined hand gestures for numbers from zero to nine.

Figure 5 shows the 10 kinds of hand gestures to be recognized in this work, from left to right, respectively, representing the digital numbers from zero to nine. To collect their depth images, ten students are invited to perform those gestures before the Kinect sensor at about three different positions, say 80 cm, 120 cm and 150 cm considering the effective range of the sensor. Their hands are placed in the front of their body for the ease of hand region segmentation. Each kind of hand gestures is repeated 20 times by one person. In this manner, the experimental dataset contains a total of 2000 samples.

### 5.2. Hand Region Segmentation and Feature Extraction

**Figure 6:**
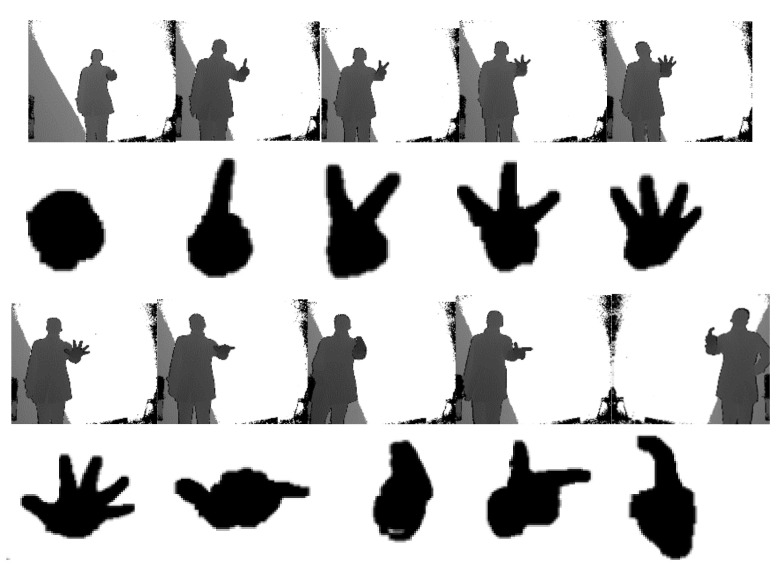
One group of depth images and segmented hand region: the depth images given in the first and third rows with corresponding hand in the second and fourth rows.

As the depth value instead of color information is used in the hand region segmentation, it is comparably easy to distinguish the hand gesture from its environment in the depth image. Figure 6 shows the depth images of one group of gestures followed by their hand regions segmented by the method suggested in Section 2.1. It can be observed from these figures that the interior of the regions is relatively uniform, and its boundary is very smooth. Only a very small empty hole is found in the region of gesture seven, and few noisy branches are kept. Therefore, the suggested hand segmentation algorithm works well.

### 5.3. Invariants of Modified Hu Moments

**Figure 7:**
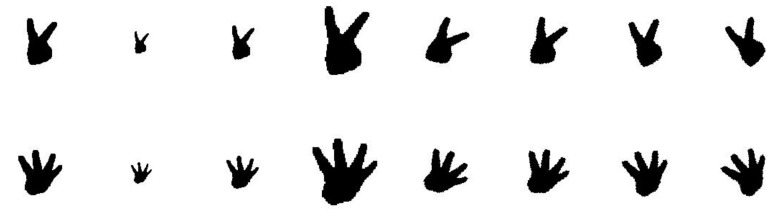
The gestures with their scale and rotation transformations.

**Figure 8:**
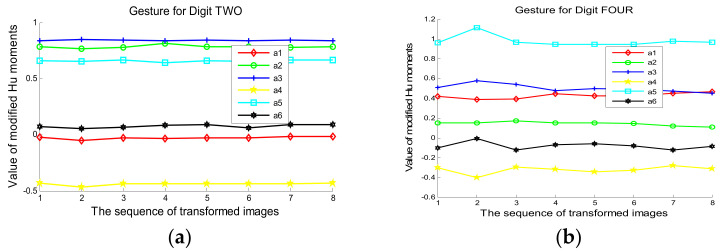
Distributions of the Hu moments via different transformations, in which the elements of a1–a6 are computed by the Formula (9): (**a**,**b**) respectively present the values of Hu moments for gestures of Two and Four.

This section validates the rotation and scale invariants of the modified Hu moments as its translation invariant is apparent. The first column in Figure 7 shows the depth images of two different gestures, while the second to fourth columns present their scales by 0.5, 0.75, and 1.5, and the last four columns demonstrate their rotations of 30° and 15° in clockwise as well as anti-clockwise motions. Figure 8 gives the estimations of six elements in the modified Hu moments with Formula (9), where the left and right figures, respectively, mark the results from the first and second gestures. It is observed that these estimations are fairly steady against those transformations, with low standard deviations of 0.0069 and 0.0278. This validates the rotation and scale invariants of the modified Hu moments both qualitatively and quantitatively. To demonstrate their discrimination ability, Table 1 presents the Euclidean distances of the modified Hu moments from those pairwise images whose order numbers are marked from #1 and #2 to #16. Strong discrimination ability can be observed from this table since the first eight images and the remainders belong to two different categories.

### 5.4. Discrimination of Confused Gestures

**Figure 9:**
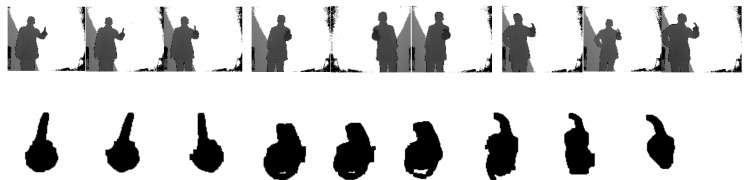
Nine samples from three different types of gestures and extracted hand regions.

**Figure 10:**
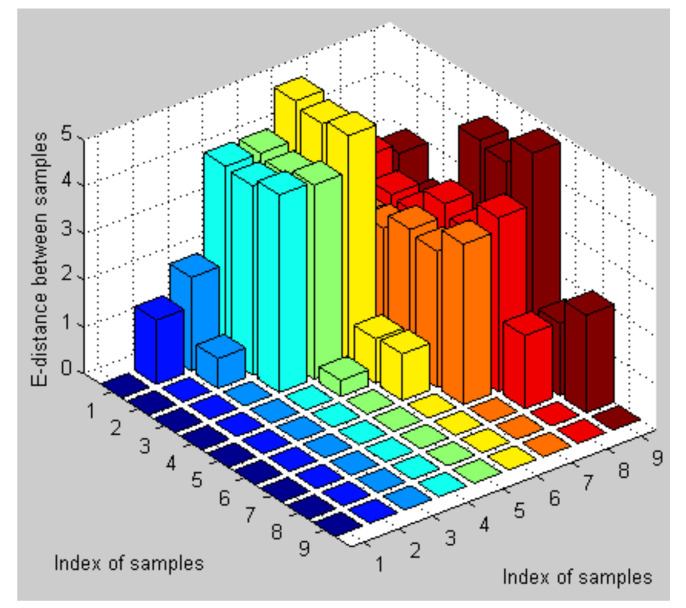
Larger interdistances and smaller intradistances among hand gestures.

Generally speaking, the smaller the distance between the gesture performer and the Kinect sensor, the bigger the image size of the hand region and the larger the distance value extracted from the fingertip to the palm center, and vice versa. In order to overcome this influence, the FGFF descriptor for each hand gesture is normalized with its $M_{min}$. To validate the discriminative ability of the weighted AdaBoost classifier for confused gestures, the feature descriptors for gesture 1, gesture 7, and gesture 9 are firstly studied. Figure 9 shows three different samples for each type of gestures and the results of hand region segmentation. With the proposed fingertip extraction procedure, just one fingertip is extracted from each of those gesture images, corresponding to one finger clustering information. The thickness of gesture 7 is obviously larger than those of the other two. Therefore, its weight factor of is bigger and the finger-earth mover’s distance method exhibits a strong distinguishing ability for gesture 7 while it is weak for the remaining two gestures since their weight factors are close to each other. In this case, the SVM model with modified Hu moments shows its advantage. For better visualization, Figure 10 shows the Euclidean distance between the Hu moment features of the nine gesture samples. It can be seen from this figure that the interdistances between different gestures are larger compared with those intradistances. This demonstrates strong recognition ability of the support vector machine and hence the weighted AdaBoost classifier.

### 5.5. Gesture Recognition and Analysis

We firstly test the effect of different volumes of training dataset on the performance of the proposed algorithm, where its training samples were randomly chosen to keep consistent distribution, varying from 25% to 75% of the whole dataset. The remainders are used as the testing samples. Table 2 gives the accuracy of hand gesture recognition versus volumes of training dataset. From this table, we can see that the accuracy is increased with the increasing volume and an acceptable balance is obtained when a half dataset is used for training the proposed classifier.

Different distances between the performer and the Kinect sensor will affect the size of hand region and produce a scale in the depth image. Subsequently, the accuracy of image processing and gesture recognition are affected. In order to validate the robustness of the weighted AdaBoost classifier against different scales, we test it under different distances. Here, thirty samples, respectively, from 80 cm distance and 120 cm distance together with forty samples from 150 cm distance were randomly chosen for testing and the rest for training. Table 3 shows the recognition results of those gestures where the last row gives average accuracy.

As can be seen from Table 3, the average recognition accuracy for the hand gestures is above 94%. Among them, the accuracy for gesture zero is always the highest, which can be correctly recognized in all tests, since this is the simplest gesture and the performer can make this action more easily and accurately. The procedure for its image segmentation and feature extraction is also the most stable and reliable. The recognition accuracy for the other hand gesture is slightly lower, but more than 92%. Better performance is observed when the distance between the gesture executor and the Kinect is positioned at about 120 cm. With the increasing distance, the recognition accuracy decreases slightly. This is because the hand region in the depth image becomes smaller, which brings the difficulty of image processing and hand region segmentation.

**Figure 11:**
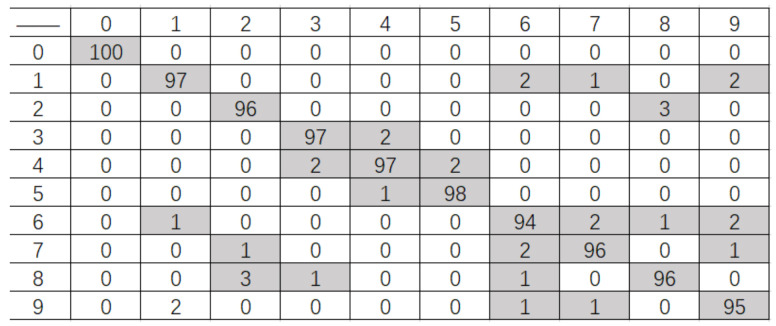
Digital gesture recognition confusion matrix.

Figure 11 shows the confusion matrix of recognition for these ten hand gestures to go into some details. As can be seen from this figure, the hand gestures for six, seven, and nine are easy to be confused. The recognition accuracy for gesture six is the lowest, which is due to the fact that the little finger in this gesture is easily overlapped in some viewpoints. This may lead to confusion with gesture one or gesture seven since their thicknesses are somewhat near to each other. For gesture seven, its degree of aggregation and curvature of the fingers is different from one performer to another, which makes the feature extraction of fingertips a bit more difficult. In this case, the weighted AdaBoost classifier shows its advantages against any single classifier. The degree of curvature in gesture nine is also performer-dependent and prone to be affected by different viewpoints, which may lead to confused recognition.

### 5.6. Comparison with Benchmark Algorithms

For comparison of recognition performance, we implement the proposed algorithm and three benchmark methods, including Ren [23], Huang [24], and Pu Xingcheng [28] as they follow a similar mechanism. To be a fair game, all the experiments are carried out on the same dataset collected in our lab. The finger-earth mover’s distance method with time-series curve representation of the hand contour was suggested in [23], while similarity measures through the Mahalanobis distance among the Hu moment features were used in [28]. In [24], different scales of circle regions centered at each of the contour points were employed to extract the area, major segment, and distance information as characteristics of the hand gesture. Basically, it is a multi-resolution analysis along the hand contour. Different ranges of finger motion as well as the noise on the contour have a considerably negative effect on the algorithm. For comparison, the FGFF descriptor and modified Hu moment in the proposed algorithm are independently tested, followed by their combinations with the weighted AdaBoost classifier, as given in the fifth, sixth, and eighth rows of Table 4. It is seen from Table 4 that our algorithm gives the highest accuracy with lower standard deviation since both finger-related and contour-related information is employed, followed by Huang [24] with 96.1% mean accuracy and 1.8 standard deviation. Since detailed shapes of the fingers are ignored in the algorithms from [23,28], their recognition accuracy is lower with an average accuracy of 95% and 95.5%, respectively. On the whole, the proposed method overcomes the shortcomings of the Benchmark methods and can obtain higher and more stable recognition accuracy.

## 6. Conclusions

We have talked about a new hand gesture recognition algorithm based on the Kinect sensor, taking the recognition of ten digital gestures from zero to nine as an example. The region growing method with double thresholds is employed to segment the hand region from its depth image where the influence of different thresholds is discussed and oversegmentation is suggested. Then, fingertip-emphasized features including palm center and fingertips together with their orientations are estimated, followed by the finger-emphasized Fourier descriptor to construct the FGFF descriptors. The modified Hu moment invariants with much lower exponents are discussed to encode the contour structure in the hand region. Finally, a weighted AdaBoost classifier is constructed based on FEMD and SVM model to realize the hand gesture recognition. The characteristics and applicability of the classifier are analyzed and compared with the three Benchmark methods reported in the literature. The results show that the proposed algorithm outperforms those algorithms with better robustness and higher recognition accuracy. Future work includes exploring more representative features and further improving the robustness of the algorithm, developing some interesting HCI applications and deploying it on our mobile robot to perform some routine housework under the instructions of hand gestures.

## Figures and Tables

**Table 1:** Distance of modified Hu moments from pairwise images.

	#1	#2	#3	#4	#5	#6	#7	#8	#9	#10	#11	#12	#13	#14	#15
#2	0.025														
#3	0.005	0.021													
#4	0.021	0.034	0.023												
#5	0.011	0.028	0.012	0.020											
#6	0.007	0.023	0.008	0.019	0.016										
#7	0.010	0.029	0.012	0.024	0.006	0.016									
#8	0.010	0.031	0.014	0.022	0.007	0.016	0.004								
#9	0.459	0.465	0.460	0.477	0.464	0.462	0.458	0.458							
#10	0.460	0.465	0.460	0.479	0.463	0.464	0.457	0.458	0.110						
#11	0.443	0.449	0.444	0.461	0.448	0.446	0.442	0.442	0.027	0.109					
#12	0.465	0.472	0.466	0.483	0.469	0.468	0.463	0.464	0.028	0.114	0.053				
#13	0.454	0.460	0.455	0.472	0.458	0.457	0.452	0.453	0.031	0.102	0.050	0.021			
#14	0.458	0.464	0.459	0.476	0.463	0.461	0.457	0.457	0.019	0.108	0.041	0.017	0.014		
#15	0.490	0.496	0.491	0.508	0.495	0.493	0.489	0.489	0.033	0.126	0.053	0.040	0.053	0.041	
#16	0.494	0.500	0.495	0.512	0.499	0.497	0.492	0.493	0.043	0.123	0.069	0.030	0.044	0.038	0.028

**Table 2:** The recognition accuracy vs. different volumes of training dataset (%).

Volumes	0	1	2	3	4	5	6	7	8	9	AVG acc.
25%	100	89.3	91.3	92.6	92.0	94.0	90.0	94.0	91.3	90.6	92.5
40%	100	93.3	94.1	95.8	96.7	96.7	94.1	96.7	95.8	93.3	95.6
50%	100	96.7	96.0	97.0	98.0	98.0	97.3	96.0	96.0	97.0	97.2
75%	100	96.7	97.3	98.6	98.0	98.0	97.3	96.7	97.0	98.0	97.7

**Table 3:** The recognition accuracy vs. different distances from the sensor (%).

Distances	0	1	2	3	4	5	6	7	8	9	AVG acc.
80 cm	100	96.7	96.7	96.7	96.7	96.7	93.3	93.3	96.7	96.7	96.3
120 cm	100	100	96.7	96.7	100	100	96.7	96.7	96.7	96.7	98.0
150 cm	100	95	95	97.5	95	97.5	92.5	97.5	95	92.5	95.7
AVG.acc	100	97	96	97	97	98	94	96	96	95	--

**Table 4:** Comparison of the proposed algorithm with Benchmark methods on our dataset (%).

Different Gestures	0	1	2	3	4	5	6	7	8	9	AVG acc.
Ren [23]	100	96	93	95	94	96	93	95	93	95	95.0
Huang [24]	100	96	96	95	96	98	94	96	96	94	96.1
Pu [28]	99	95	96	96	95	97	94	94	95	94	95.5
FGFF+FEMD	100	96	94	96	95	96	94	94	95	94	95.4
Hu+SVM	98	96	96	96	95	97	93	95	96	95	95.7
Proposed Algorithm	100	97	96	97	97	98	94	96	96	95	96.6

## Data Availability

The data presented in this study are available on request from the corresponding author.
